# Invention of Artificial Rice Field Soil: A Tool to Study the Effect of Soil Components on the Activity and Community of Microorganisms Involved in Anaerobic Organic Matter Decomposition

**DOI:** 10.1264/jsme2.ME20093

**Published:** 2020-09-19

**Authors:** Yu Maeda, Kazumori Mise, Wataru Iwasaki, Akira Watanabe, Susumu Asakawa, Rasit Asiloglu, Jun Murase

**Affiliations:** 1 Graduate School of Bioagricultural Sciences, Nagoya University, Furo-cho, Chikusa-ku, Nagoya 464–8601, Japan; 2 Graduate School of Science, The University of Tokyo, Yayoi, Bunkyo-ku, Tokyo 113–0032, Japan; 3 Faculty of Agriculture, Niigata University, Niigata 950–2181, Japan

**Keywords:** HTS, humus, methane, paddy soil, soil microcosm

## Abstract

Soils are characterized by diverse biotic and abiotic constituents, and this complexity hinders studies on the effects of individual soil components on microorganisms in soil. Although artificial soils have been used to overcome this issue, anoxic soils have not yet been examined. We herein aimed to create artificial soil that reproduces anaerobic methane production by soil from a rice field. Organic materials and mineral particles separated from rice field soil were mixed to prepare an artificial soil matrix; the matrix was added with a small volume of a soil suspension as a microbial inoculum. When the microbial inoculum was added immediately after matrix preparation, anaerobic decomposition was markedly less than that by original soil. When the inoculum was added 9–15 days after soil matrix preparation, anaerobic CO_2_ and methane production was markedly activated, similar to that by original soil after 40 days of incubation, which suggested that the maturation of the soil matrix was crucial for the reproduction of anaerobic microbial activities. The diversity of the microbial community that developed in artificial soil was markedly less than that in original soil, whereas their predicted functional profiles were similar. Humic substances altered the composition and network patterns of the microbial community. These results suggested that the functional redundancy of soil microorganisms was sustained by different microbial sub-communities. The present study demonstrated that artificial soil is a useful tool for investigating the effects of soil components on microorganisms in anoxic soil.

Soil is one of the most complex microbial habitats on Earth. Soil microorganisms play an essential role in soil ecosystem services, interacting not only with other organisms, but also with organic and inorganic soil components (Voroney and Heck, 2015). The effects of soil abiotic factors on the ecology of microorganisms are a central question in soil microbiology. However, due to the complexity of soil, difficulties are associated with elucidating the individual effects of its specific components on the microbial community.

Model soil ecosystems have often been used to experimentally investigate the impact of the various components of different soils. One of the earliest and simplest models consisted of an anion-exchange resin, which was used to examine physiological changes in bacteria adsorbed to a soil-like solid surface (*e.g.*, Hattori *et al.*, 1972). Simple materials, such as sand and vermiculite, have also been used to investigate plant-microbe interactions and the effects of microfauna (*e.g.*, Brimecombe *et al.*, 1999; Simon *et al.*, 2001). An alternative approach is the use of artificial soil systems containing all of the essential chemical components of soil, but with reduced heterogeneity and biological complexity. These models are valuable for analyzing many of the abiotic and biotic processes of soil. Artificial soils developed by Ellis (2004) to examine microbial autoecology were made of sand, clay minerals, calcium carbonate, and humic acids. In another study, the effects of the clay mineral type on the temperature sensitivity of cellulose and lignin decomposition were demonstrated using artificial soils inoculated with a mixture of the natural microbial communities extracted from soils from local forest, prairie, and wetland sites (Zhang *et al.*, 2011). Guenet *et al.* (2011) proposed a protocol for artificial soil containing different aggregate sizes to achieve a more realistic approximation of natural soil. In a series of artificial-soil maturation experiments, the types and amounts of clays, metal oxides, and charcoal were shown to play pivotal roles both in the formation and persistence of soil aggregates and elucidation of microbial compositions and enzymatic activities (Pronk *et al.*, 2017 and references therein). Despite the successful applications of artificial soils, it currently remains unclear whether artificial soils reflect the microbial activities of natural soils.

Wetland rice soils are characterized by biogeochemical cycles and microbial communities that differ from those in upland soils (Kimura, 2000; Conrad and Frenzel, 2002). Processes unique to wetland soils include the methanogenic degradation of organic matter, which is accomplished by a‍ ‍complex community of anaerobic microorganisms comprising primary and secondary fermenters, syntrophic species, and methanogens that participate in the exchange of substrates and electrons (Schink, 1997). Different environmental factors, such as drainage, organic amendment, fertilization, temperature, and plants, have been shown to affect methane production and the methanogenic microbial community in wetland rice soils (Conrad, 2007). In contrast, the effects of individual soil components on methanogenic microbial consortia remain largely unknown; no artificial systems of anoxic soils have been proposed.

Humic substances are essential constituents of soil because they play not only a static role in carbon storage, but also dynamic roles in terrestrial ecosystems. Reductive and oxidative reactions and the sorption, complexation, and stabilization of the soil structure are some of the processes in the soil environment that involve humic substances (Lipczynska-Kochany, 2018). Humic and fulvic acids adsorb on clay minerals to form humus-clay complexes (Zhang *et al.*, 2012). By acting as electron donors, acceptors, and shuttles, humic substances enhance anaerobic microbial metabolism (Lipczynska-Kochany, 2018). Klüpfel *et al.* (2014) reported that humic acids from natural environments function as renewable electron acceptors for the anaerobic growth of *Shewanella oneidensis*. Conversely, due to their high content of stable free radicals (Paul *et al.*, 2006), they may also inhibit bacterial enzymes (Fujimura *et al.*, 1994; Van Trump *et al.*, 2006; Mazzei *et al.*, 2013). Therefore, humic substances may be a key factor that controls the performance and community of microbial guilds involved in anaerobic organic decomposition in wetland soils; this question may be analytically addressed using artificial soil.

In the present study, we aimed to invent artificial soil that reproduces methanogenic organic matter decomposition by soil from a rice field. To achieve this, the constituents of artificial soil, including organic matter, mineral particles, and soil microorganisms, were extracted from original soil and then remixed. We assumed that the different microbial communities out of the “seed” community will proliferate in artificial soil under different conditions. The present study also investigated whether the maturation of artificial soil affects the performance and community structure of microbes, assuming the stabilization of mixed abiotic constituents as a key for the successful development of wetland artificial soil. Moreover, as a case study to examine the effects of soil components on microorganisms in anoxic artificial soils, the impact of humic substances was investigated by comparing the activities and community of microorganisms in artificial soils with and without humic substances.

## Materials and Methods

### Soil sample

A yellow soil (Oxiaquic Dystrochrepts) was sampled in February, 2010 (off-crop season) from a rice field at the Anjo Research and Extension Station, the Agricultural Research Center of Aichi Prefecture, Central Japan (34°58'21"N, 137°4'34"E). The characteristics of air-dried soil were as follows: pH 6.3 (1:1, soil/water ratio), cation-exchange capacity of 14.4‍ ‍cmol kg^–1^, organic C content of 13.1‍ ‍g kg^–1^, total N content of 1.62‍ ‍g kg^–1^, and clay content of 23% (Lu *et al.*, 2002). This soil was passed through a sieve (<2‍ ‍mm) and stored at 4°C until used.

### Isolation of soil constituents

#### Minerals

Mineral particles in soil were obtained by hydrogen peroxide digestion during heating on a hot plate (Day, 1965). Soil mineral suspensions that remained after the digestion were collected by centrifugation (10,000×*g*, 10‍ ‍min), washed three times with pure water, and dried at 105°C for 24 h. They were then weighed, homogenized with a mortar and pestle, and heat-sterilized at 160°C for 2 h.

#### Humic substances

Humic and fulvic acids were extracted from soil, purified, and freeze-dried as described previously (Kuwatsuka *et al.*, 1992). In brief, humic and fulvic acids were extracted with 0.1 M NaOH. Crude humic acids in the extract were deposited by acidification with 3 M HCl at pH 1.0 and the supernatant solution was transferred for the preparation of fulvic acids. Humic acids were prepared through the repeated processes of neutralization, dissolution, and reprecipitation, and then freeze-dried. The fulvic acid fraction was neutralized, desalted, acidified, and then freeze-dried. The carbon content of humic substances was assessed using a total organic carbon analyzer (TOC-V_CPH_; Shimadzu) after dissolution.

#### Plant residues

Soil was suspended in cold (4°C) distilled water and serially passed through two sieves (1- and 0.5-mm mesh sizes). The plant residues retained on the sieves were manually collected using tweezers, dried at 50°C for 48 h, and then autoclaved.

#### Microbial inoculum

The soil suspension was used to inoculate a natural assemblage of microorganisms in anoxic soil. A mixture containing 150‍ ‍g of rice field soil and pulverized rice straw (*Oryza sativa* L. var. Nipponbare; 0.6%, w/w) was incubated under flooded conditions in a 200-mL beaker at 25°C. After 2‍ ‍weeks of incubation, the anoxic soil layer was collected using a truncated glass syringe (volume of 25‍ ‍mL) and mixed with O_2_-free sterilized water under anoxic conditions, yielding a 40-fold diluted soil suspension.

### Maturation and incubation of artificial soils

Artificial soil was prepared by mixing the extracted soil components at ratios based on the analytical results of original soil: 5‍ ‍g of minerals, 0.1‍ ‍mg of KNO_3_, 0.4‍ ‍mg of CaSO_4_, 13 (0.5–1‍ ‍mm size) and 19 (1–2‍ ‍mm size) mg of plant residues, 7.4‍ ‍mgC of fulvic acids, and 5.4‍ ‍mgC of humic acids. KNO_3_ and CaSO_4_ solutions were added as supplemental ions because both were mostly lost during the preparation of soil components. The amounts of fulvic and humic acids were considered to be similar to those in original soil based on previous findings (Watanabe and Kuwatsuka, 1991). Pulverized rice straw (30‍ ‍mg) was added as a carbon source for methanogenesis because the soil used in the present study only produced a small amount of methane during the anoxic incubation without the added organic materials (Murase and Kimura, 1994). The diluted soil suspension (5‍ ‍mg, dry weight) was added to artificial soil as a microbial inoculum immediately after the other soil components had been mixed; we refer to this microcosm as “Humus 0”. Our pilot experiment revealed the poor microbial activity of “Humus 0” soil; therefore, subsets of artificial soils were inoculated with a diluted soil suspension (inoculum) after 9 and 15 days of mixing the other soil components (referred as “Humus 9” and “Humus 15”) in order to investigate the effects of artificial soil maturation on the microorganisms inoculated thereafter. We assumed that the maturation process included the adsorption of fulvic and humic acids on clay minerals, which was completed in 3 days (Zhang *et al.*, 2012; Pronk *et al.*, 2017); maturation days (9 and 15 days) were selected to ensure the adsorption process. To examine the effects of humic substances on the performance and community of microbial populations, artificial soil without humic substances was also prepared and immediately inoculated with a diluted soil suspension (referred to as “Humus –”).

Artificial soil samples were prepared in triplicate, water-saturated, and incubated in 30-mL Gas-Chro vials (Nichiden-Rika Glass) under anoxic conditions at 25°C. Original soil supplemented with rice straw was similarly prepared for comparisons.

### Gas analysis

The concentrations of CH_4_ and CO_2_ in the headspace of the vials were measured using a gas chromatograph equipped with a flame ionization detector and thermal conductivity detector (GC-14B; Shimadzu).

### DNA extraction and amplicon sequencing of 16S rRNA genes

DNA was extracted from 0.5‍ ‍g of soil samples after 66 days of incubation using ISOIL for Beads Beating (Nippon Gene). Extracted DNA was purified using the OneStep^TM^ PCR inhibitor removal kit (Zymo Research) and quantified using the Quant-iT PicoGreen dsDNA assay kit (Invitrogen).

To characterize the diversity and composition of the soil bacterial and archaeal communities in each sample, the V3/V4 regions of the 16S rRNA genes from each soil extract were amplified using the primer pair 341F (5′-TCGTCGGCAGCGTCAGATGTGTATAAGAGACAGCCTACGGGNGGCWGCAG-3′) and 785R (5′-GTCTCGTGGGCTCGGAGATGTGTATAAGAGACAGGACTACHVGGGTATCTAATCC-3′), which provides good coverage for prokaryotes and was designed for optimal use on Illumina MiSeq DNA sequencing platforms (Illumina) (Klindworth *et al.*, 2013). Primers included the Illumina adapter sequences (underlined) required for downstream sequencing. The PCR mixture contained 12.5‍ ‍ng of DNA, 5‍ ‍μL of 1‍ ‍μM each primer, and 12.5‍ ‍μL of 2× KAPA HiFi HotStart ReadyMix (KAPA Biosystems) in a total volume of 25‍ ‍μL. PCR amplification was conducted in triplicate for each soil sample under the following conditions: (i) 95°C for 3‍ ‍min; (ii) 25 cycles of 95°C for 30‍ ‍s, 55°C for 30‍ ‍s, and 72°C for 30 s; and (iii) 72°C for 5‍ ‍min. PCR products were purified using Agencourt AMPure XP beads (Beckman Coulter) and then mixed in equal amounts after quantification using the Quant-iT PicoGreen dsDNA assay kit. The mixture of PCR products was then sequenced on an Illumina MiSeq instrument using 2-by-300-bp chemistry. Prior to DNA sequencing, the sequencing provider attached a unique combination of Nextera XT dual indices (Illumina) to the DNA from each sample to allow for multiplex sequencing. Data have been deposited with links to BioProject accession number PRJDB8477 in the DDBJ BioProject database.

### Bioinformatics analysis

Raw sequences were processed using the Quantitative Insights Into Microbial Ecology (QIIME) toolkit (Caporaso *et al.*, 2010). Primers, barcodes, sequences with a quality score <25, and sequences <200 bp or containing any unresolved nucleotides were excluded from the analysis using the default settings. The remaining sequences were denoised, followed by chimera identification and removal via ChimeraSlayer. In total, 1,453,702 high-quality sequences were obtained from all 15 samples (67,367–120,019 sequences per sample; mean 98,307). After the removal of singletons, sequences with 97% similarity were assigned to OTUs, yielding 17,497 OTUs. The rarefaction curve for the observed number of OTUs was calculated to assess the richness of the bacterial and archaeal 16S rRNA gene sequences in the samples. Unweighted and weighted UniFrac matrixes (Lozupone and Knight, 2005) of samples were generated by QIIME and subjected to statistical analyses. Taxonomic information was assigned using the NGS analysis pipeline of the SILVA rRNA gene database project (SILVAngs 1.3) and the default pipeline parameters (Quast *et al.*, 2013).

Co-occurrence networks based on the abundance of 16S rRNA gene OTUs were constructed for artificial soils with and without humus—neither had any maturation period (Humus 0 and Humus‍ ‍–)—and original soil by following the pipelines developed by Williams *et al.* (2014). The OTU table at the genus level was rarefied to a sample depth of 60,000 covering all three soils, and co-occurrence between all pairs of prokaryotes was evaluated using Spearman’s correlation coefficient in R (*P*<0.05). Positive (ρ=1) and negative (ρ=–1) correlations were screened out and co-occurrence networks were visualized for each sample type in Cytoscape software v. 3.7.2 using the compound spring embedded layout (Shannon *et al.*, 2003).

Functional pathways and marker genes were predicted using an OTU table generated from QIIME, PICRUSt (Langille *et al.*, 2013), the Kyoto Encyclopedia of Genes and Genomes (KEGG) orthology (Kanehisa *et al.*, 2004), and the Cluster of Orthologous Genes (COG). Chimera-checked sequences were subjected to‍ ‍closed-reference OTU picking against Greengenes 13_5 (McDonald *et al.*, 2012), using QIIME and the default parameters. The OTU table was normalized to the 16S rRNA gene copy number per cell; therefore, the abundance of each OTU more accurately reflected the number of microbes in each OTU. The normalized OTU table was then used to convert 16S rRNA gene sequencing data into a predicted metagenome for each sample according to the reference database (Greengenes 13_5). The reference genome coverage for each sample was calculated and presented as the Nearest Sequenced Taxon Index (NSTI). After a predicted metagenome had been created using PICRUSt, the biological pathway and functional gene composition of each sample were estimated based on the KEGG orthology (KEGG pathway level 2) and COG classification scheme. In addition, the relative abundance of KEGG orthology (KO) groups assigned to hydrogen production (hydrogenase), acetate metabolism (acetate kinase and acetyl-CoA synthase), and methane oxidation (methane monooxygenase), which were detected in the rice field soils (Masuda *et al.*, 2018), was calculated. Methyl-coenzyme M reductase (mcrA) as not included in the analysis because Greengenes used in PICRUSt is less reliable for the classification of Archaea due to the smaller number of reference sequences in the database than in SILVA.

### Statistical analysis

The significance of differences in CO_2_ and methane production as a function of the microbial community, defined in terms of changes in the number of OTUs and NSTI values between samples, was tested using one-way and multiple-comparison ANOVAs (SPSS 22.0). Multivariate statistical routines were conducted using PRIMER 7 and PERMANOVA+ (Primer-E) (Clarke and Gorley, 2015). A principal coordinates analysis (PCoA) was performed based on unweighted and weighted UniFrac distances. The predicted KEGG pathways and COGs were subjected to a cluster analysis based on the Bray-Curtis similarity and using the group average method. An ANOSIM (analysis of similarity) was performed to test the null hypothesis that there was no difference in the microbial communities and predicted functional profiles between treatments, based on pairwise comparisons.

## Results

### CO_2_ and methane production in artificial soils

**Fig. 1.**
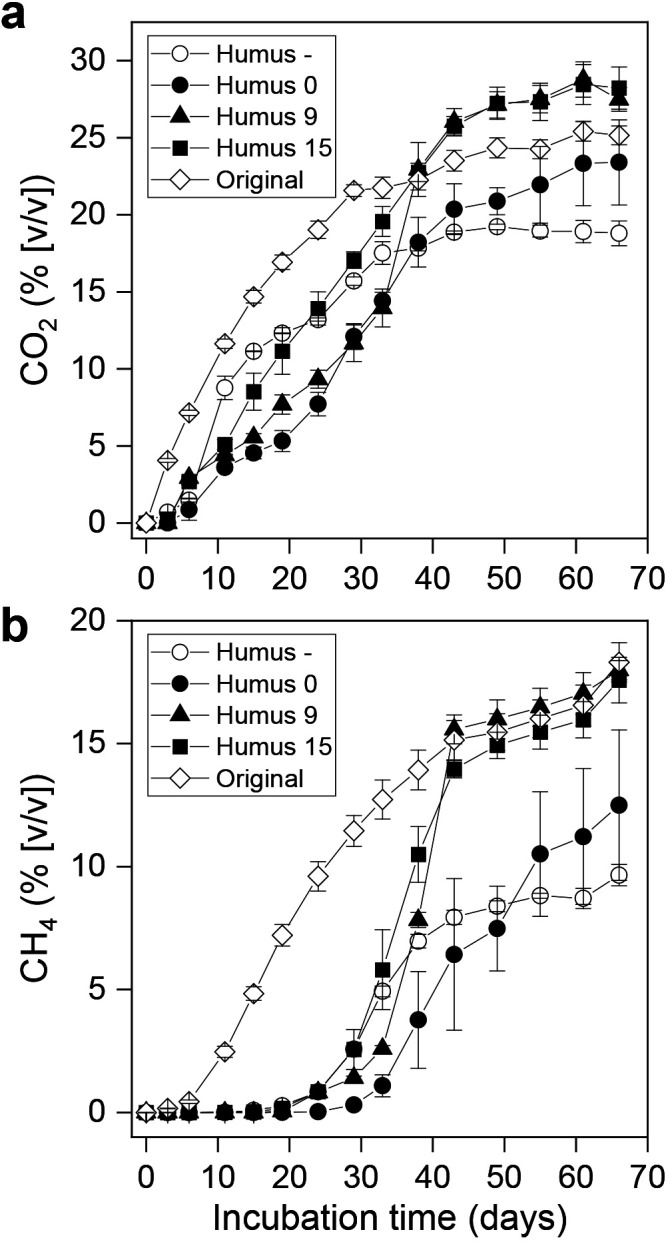
Concentrations of (a) CO_2_ and (b) methane in the headspace of incubation vials containing artificial soils. The error bars represent the standard error (*n*=3). Humus –: artificial soils with no humic substances added; Humus 0, 9, 15: artificial soils containing humic substances and inoculated with a microbial suspension after 0, 9, and 15 days of an artificial soil preparation. Original, original rice field soil.

CO_2_ production was detected in all artificial soils immediately after the start of the anoxic incubation; however, the initial increase was lower than that measured in original soil (Fig. 1a). Artificial soils allowed to mature for 15 days before the microbial inoculation (Humus 15) produced more CO_2_ than those without maturation (Humus 0) and a shorter maturation time (Humus 9). Humus 9 samples reached the same level of CO_2_ production as Humus 15 samples after 40 days, whereas CO_2_ production by Humus 0 samples was lower than that by Humus 9 and Humus 15 samples. In all artificial soils supplemented with humic substances, CO_2_ production by the end of the incubation was similar to or higher than that by original soil.

Artificial soil without added humic substances (Humus –) initially produced more CO_2_ than artificial soil containing humic substances and soil microbes added at the same time (Humus 0); however, CO_2_ production in the former stopped after 40 days of incubation. In contrast, CO_2_ production continued in Humus 0 samples and, after 40 days of incubation, the amount was larger than that in Humus – samples.

Methane production by artificial soils, except Humus 0, was detected after 11 days of incubation, although initial production was markedly less than that by original soil, which actively produced methane after 6 days of incubation. Active methane production by artificial soils was observed after 30 days (Fig. 1b). In Humus 9 and Humus 15 soils, methane production reached a similar level to that in original soil after 43 days of incubation. Methane production by Humus 0 samples was markedly less than that by Humus 9 and Humus 15 samples. Humus – samples showed earlier methane production than Humus 0 samples, but stopped production after 43 days of incubation, which was similar to Humus 0 samples.

A comparison between humus-supplemented artificial soils showed that a longer maturation period before microbial inoculation resulted in an earlier and/or higher level of production of CO_2_ and methane.

### Microbial community in artificial soils

**Fig. 2.**
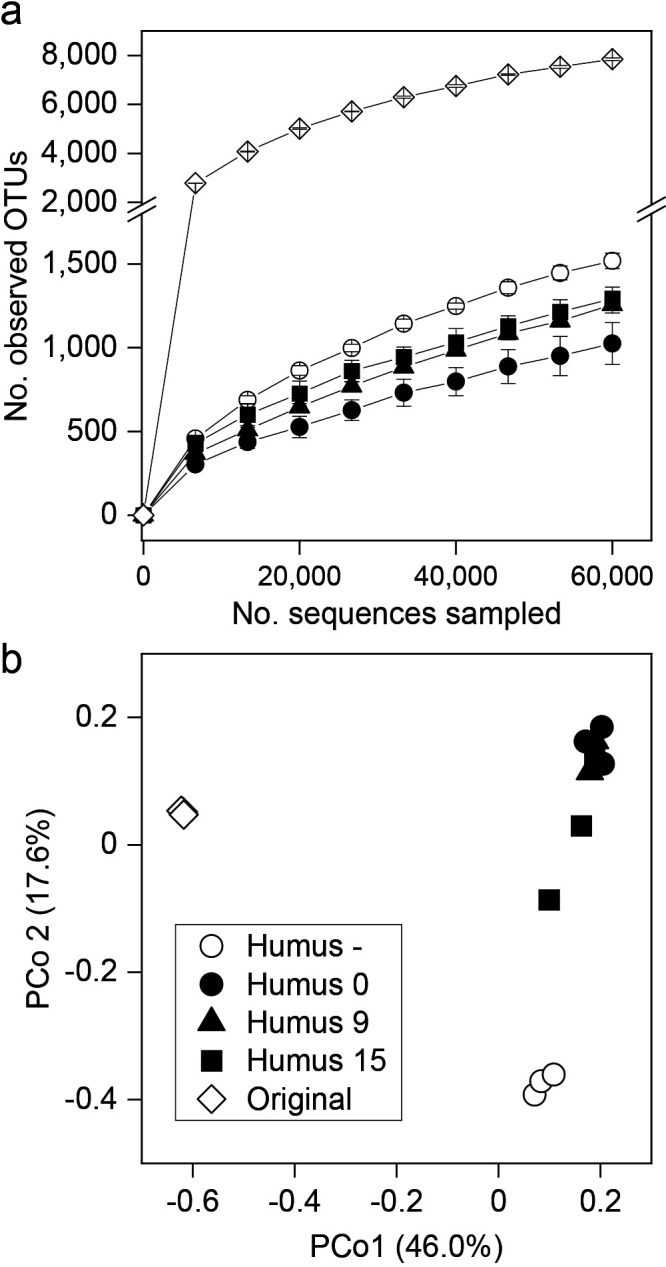
(a) Rarefaction curve and (b) principal coordinate analysis (PCoA) based on weighted UniFrac of microbial communities of artificial soils developed after the incubation experiment.

The diversity of the prokaryotic community that developed after the course of the incubation in artificial soils was markedly less than that in original soil in terms of the number of OTUs at the highest number of sequences sampled (60,000) (Fig. 2a, *P*<0.001). The maturation of artificial soils had no significant effect on microbial diversity. Soil without humic substances (Humus –) had a higher number of OTUs than Humus 0 soil (*P*=0.006).

PCoA based on the unweighted UniFrac distance similarly showed that the prokaryotic community of artificial soils was distinct from that of original soil, a result that was further supported by the results of ANOSIM (*P*<0.001, pairwise R values=1.000; Fig. 2b). The maturation of artificial soils before the microbial inoculation (Humus 9 and 15 soils) had no significant effect on the prokaryotic community from that of Humus 0 soil. On the other hand, the microbial community of Humus – differed from the communities in soils with added humic substances (pairwise R values=0.926–1.000). PCoA based on the weighted UniFrac distance gave a similar result (Fig. S1).

**Fig. 3.**
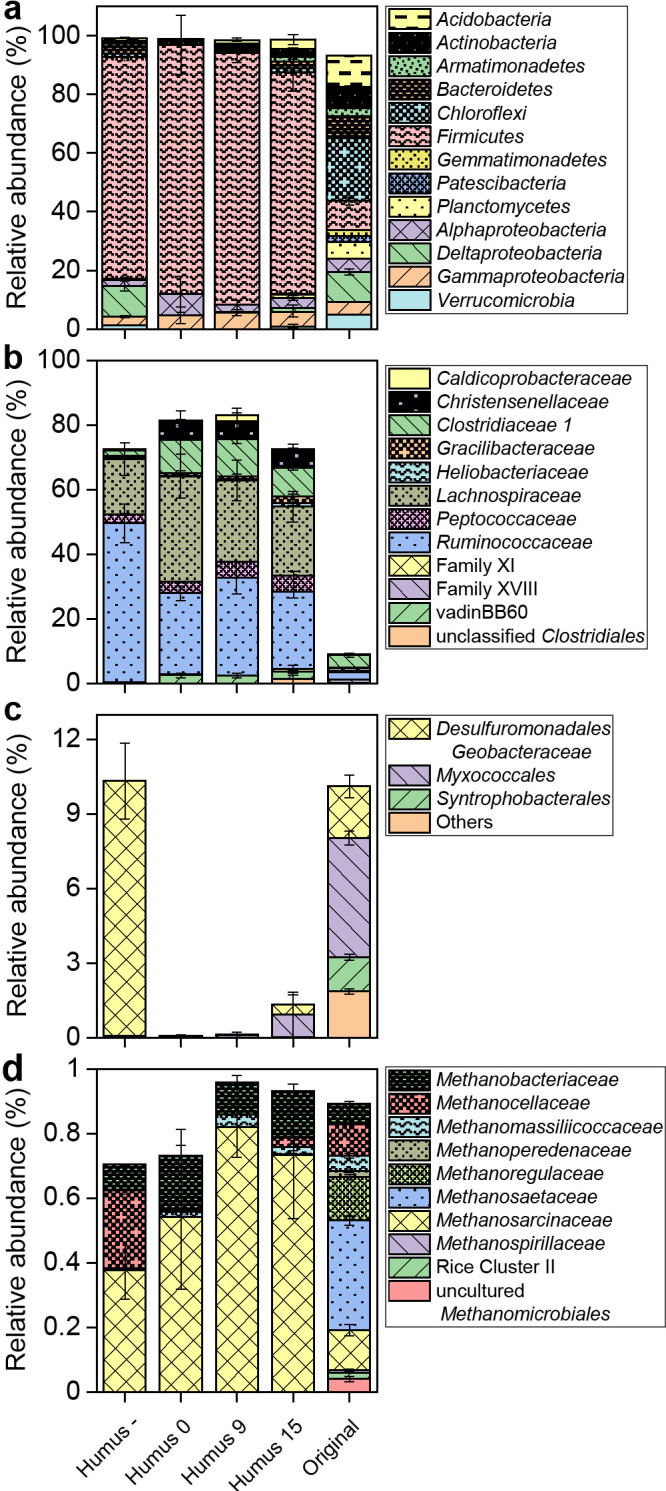
Taxonomic classification of the microbial community in artificial soils after the incubation experiment. (a) The whole community at the phylum and class (for proteobacteria) levels; (b) *Clostridiales*; (c) *Deltaproteobacteria*; (d) methanogens

The prokaryotic community of artificial soils was highly dominated at the phylum level by *Firmicutes*, followed by *Proteobacteria*, while original soil was dominated less by *Firmicutes* and included more diverse groups, such as *Chloroflexi*, *Planctomycetes*, and *Verrucomicrobia* (Fig. 3a).

Different families of *Clostridiales* dominated *Firmicutes* in artificial soils regardless of the maturation periods and addition of humic substances (Fig. 3b). In artificial soils with added humic substances, the relative abundance of *Lachnospiraceae*, *Ruminococcaceae*, and *Clostridiaceae* was high, with smaller contributions by *Christensenellaceae*, *Peptococcaceae*, and other families. Humus – soils also had *Ruminococcaceae* and *Lachnospiraceae* as the major *Clostridiales* families, but less for the other families than Humus 0.

*Alpha-* and *Gammaproteobacteria* were the dominant proteobacteria in artificial soils supplemented with humic substances (Fig. 3a), with *Micropepsaceae* and *Burkholderiaceae* as the dominant families, respectively (data not shown). In‍ ‍Humus – soil, *Deltaproteobacteria* dominated, with *Desulfuromonadales* (*Geobacteriaceae*), related to the genus *Geobacter*, accounting for a very large proportion of the population (Fig. 3c and S1).

Methanogenic archaea comprised up to 1.0% of the prokaryotic community. Ten different families of methanogens were detected in original soil, in contrast to only 3–4 families in artificial soils. *Methanosarcinaceae* and *Methanobacteriaceae* predominated and *Methanosaetaceae*, which was the most dominant group in original soil, was not detected in any artificial soils. (Fig. 3d). The effects of maturation were not obvious, while the addition of humic substances caused a shift in the methanogen community composition. Humus – soil contained a high relative abundance of *Methanocellaceae*.

### Co-occurrence patterns of the microbial community in artificial soils

Since the maturation period did not significantly affect the microbial community of artificial soils, we conducted a network analysis that focused on the difference between original and artificial soils and on the impact of the application of humic substances.

**Fig. 4.**
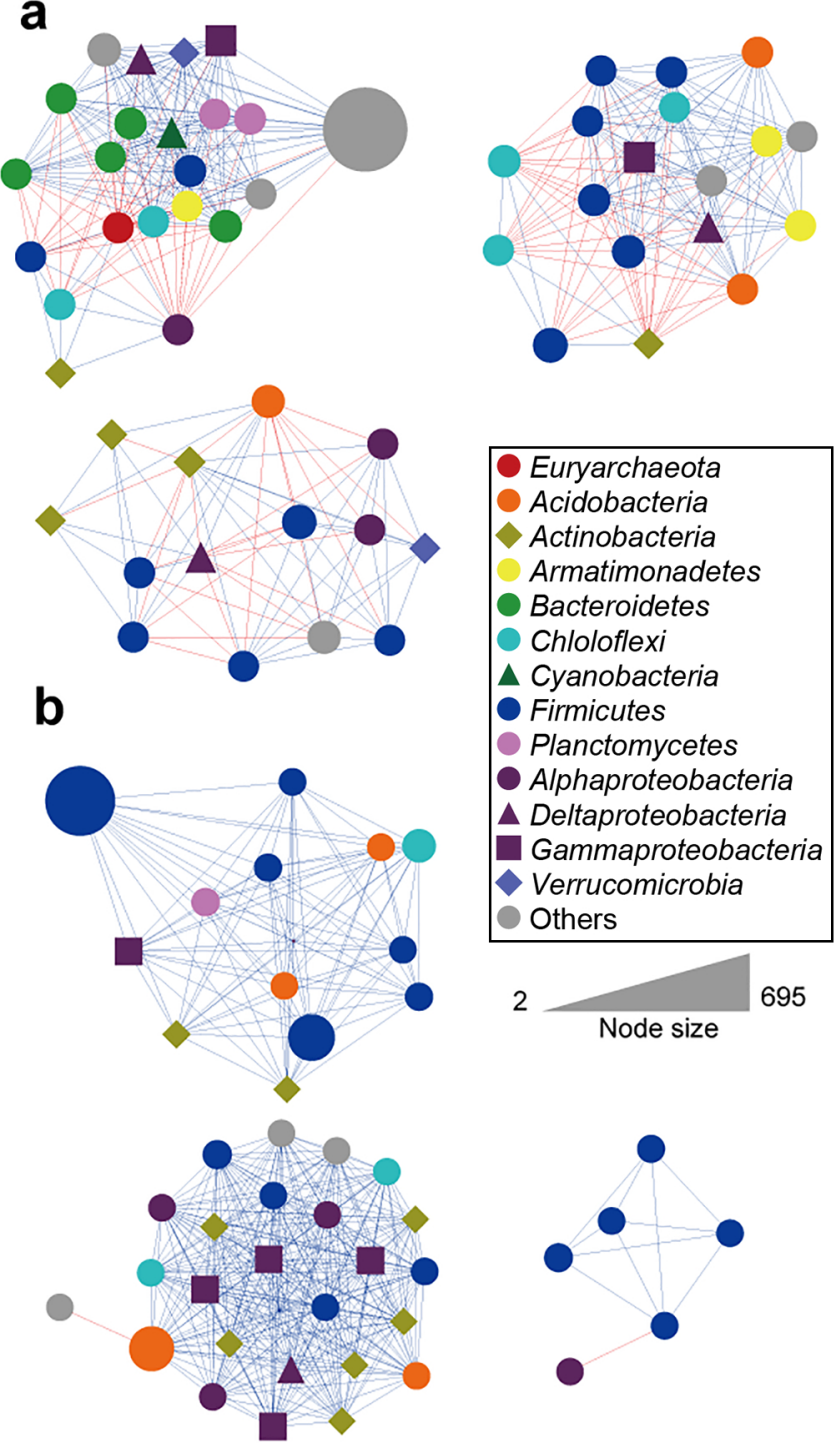
Co-occurrence networks based on a correlation analysis from genus-level taxonomic profiles of prokaryotic communities of artificial soils (a) without humus added (Humus –) and (b) with humus added (Humus 0). Node sizes indicate the mean taxonomic abundance. Positive co-occurrence correlations (ρ=1, *P*<0.05) were indicated with blue-colored edges, while negative co-occurrence correlations (ρ=–1, *P*<0.05) were indicated with red-colored edges.

Artificial soils (Humus 0 and Humus –) and original soils both exhibited 3 modules, with 46, 54, and 388 nodes being included in Humus 0, Humus –, and original soils, respectively (Fig. 4 and S2). *Firmicutes*, mostly *Clostridia*, was the predominant group in the network of artificial soils, but only one common OTU was identified among them. Furthermore, only two OTUs of *Planctomycetes* and *Gammaproteobacteria* were commonly included in the networks shared in Humus 0 and Humus – soils. The total number of significant co-occurrence relationships identified in the Humus 0 and Humus – communities were 403 and 397, which was markedly less than that in original soil: 19,645 pairwise correlations. The network in Humus 0 soil was dominated by positive relationships with only 2 negative relationships, whereas the Humus – community contained 111 negative relationships. No common relationship was identified among Humus 0 and Humus – soils.

### Predicted microbial functions in artificial soils

Most of the NSTI values were within the range of 0.17±0.02 (mean±s.d.), which indicated that the predictions of the functional gene content were informationally useful (Langille *et al.*, 2013). NSTI values were lower in artificial soils than in original soil, except in the case of Humus 15, and higher in artificial soils with than without humic substances (Fig. S3).

**Fig. 5.**
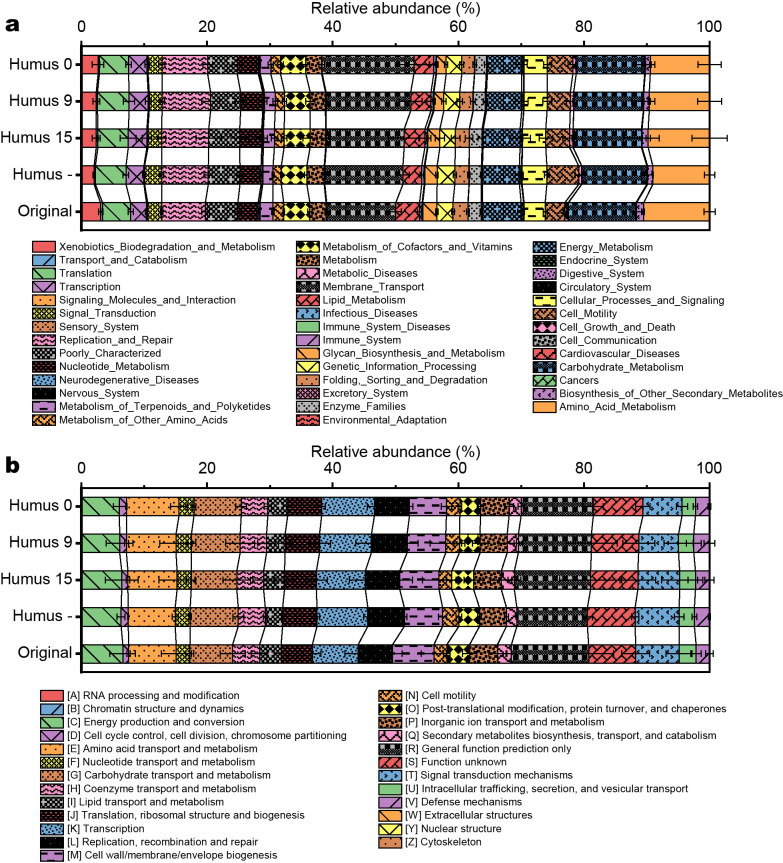
Predicted microbial functions in artificial soils. (a) Predicted KEGG pathways; (b) COG functional categories.

Every artificial soil contained the predicted KEGG pathways and COG functional categories that were similar to the suite in original soil at level 2 (Fig. 5). The Bray-Curtis similarity between samples was very high, both for the predicted KEGG pathways (91.7–99.8%) and COG functional categories (92.4–99.7%) (Fig. S4). However, in ANOSIM, the profiles of the predicted KEGG pathways of artificial soils significantly differed from that of original soil (*P*<0.001; pairwise R values=0.889–1.000). Differences were also found between Humus – soil and artificial soils with added humic substances (pairwise R values=0.704–1.000). No significant differences were observed in the profiles of the COG functional categories between the different treatments of artificial soils.

**Fig. 6.**
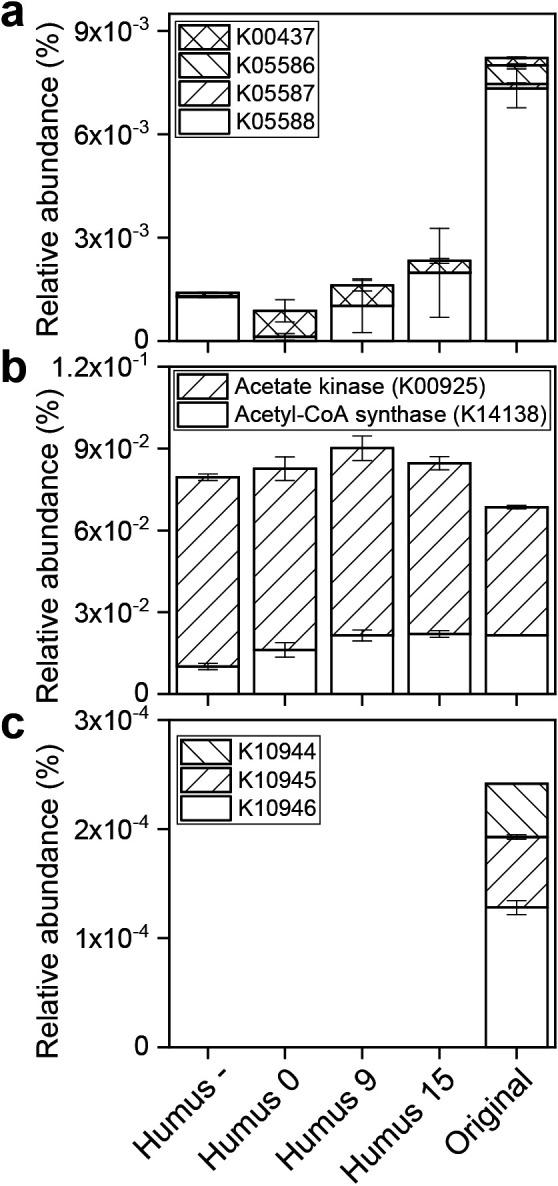
Predicted relative abundance of KEGG orthology groups associated with (a) NiFe-hydrogenase, (b) acetate metabolism, and (c) methane monooxygenase.

Maturation caused a slight shift in dominant KO assigned to hydrogenase, but not in KO assigned to genes involved in acetate metabolism (Fig. 6). Artificial soils did not include KO assigned to methane monooxygenase, most likely due to the anaerobic incubation. The dominant KO assigned to hydrogenase differed between Humus 0 and Humus –.

## Discussion

Artificial soils have been used to examine the roles of soil constituents in microbial processes. In the present study, we developed artificial soil that was representative of wetland rice field soil to reproduce methanogenic decomposition by rice straw, one of the major organic materials introduced into rice field soils (Kimura *et al.*, 2004). Artificial soils, made of soil components from a single source, enabled us to compare the microbial activities of the artificial soils developed with those of original soil and examine the effects of the specific soil component, humic substances.

The size of the microbe-containing inoculum was small: approximately 0.1% of original soil on a weight basis; however, after 40 days of the anoxic incubation, the amounts of CO_2_ and methane production in artificial and natural soils were similar (Fig. 1). These results demonstrated that the inoculated microbes colonized the artificial substrates and performed anaerobic organic matter decomposition similarly to the original system, as evidenced by the rapid increase in CO_2_ and methane production under the favorable conditions of the experiment. Angel *et al.* (2012) previously reported that upland soils, including desert soils with an undetectable level of methanogens (<10^3^ copies g^–1^), produced methane during an anoxic incubation and that methanogen numbers increased up to 10^9^ copies g^–1^ in 48 days. Thus, the inoculum size used in the present study appeared to be sufficient to reestablish the active microbial population. We consider the effects of the abiotic components included in the inoculum to be negligible because of the low mixing ratio. The longer delay in methanogenesis than in CO_2_ production suggests that the methanogenic microbial consortium, in which a suite of different functional groups of anaerobic microorganisms cooperate symbiotically (Schink, 1997), requires a longer time for its establishment than anaerobic respirators and fermenters that independently utilize organic matter to produce CO_2_.

The longer maturation period of the matrix of artificial soil before microbial inoculation resulted in more rapid and promoted anaerobic organic matter decomposition, which suggests that maturation had a strong impact on anaerobic gas metabolism in artificial soils being crucial for the successful creation of anaerobic artificial soils. The maturation process of artificial soils includes the association of organic matter with clay minerals (Heister *et al.*, 2012; Pronk *et al.*, 2012; Zhang *et al.*, 2012), which may rapidly occur (Pronk *et al.*, 2012; Zhang *et al.*, 2012). This organo-mineral association may mask or prevent the inhibitory effects of humic substances on certain microbial activities (Fujimura *et al.*, 1994; Van Trump *et al.*, 2006; Mazzei *et al.*, 2013), for example, by neutralizing free radicals (Paul *et al.*, 2006). This is supported by the result showing that the exclusion of humic substances increased gas production in the initial stage of the anaerobic incubation.

The predominance of *Clostridia* in artificial soils in the present study was consistent with previous findings of bacterial communities colonizing and decomposing rice straw in anoxic rice field soil (Weber *et al.*, 2001; Wegner and Liesack, 2016; Ji *et al.*, 2018). Thus, the microbial community that developed in artificial soils most likely represented the populations that are actively involved in the anaerobic decomposition of rice straw under the experimental conditions. Due to the lower microbial diversity of artificial soils than original soil, the present results indicate that a subpopulation out of the microbial population in the inoculum was involved, which in turn implies the functional redundancy of soil microorganisms (Nannipieri *et al.*, 2003). This was also represented by the methanogenic community that developed in artificial soils consisting of groups that were not necessarily dominant in original soil. Masuda *et al.* (2018) reported that only limited groups of methanogens inhabiting rice field soil expressed the functional gene (*mcr*) in the field at a specific time.

In contrast to the low diversity, the predicted KEGG pathways and COG functional categories of the microbial communities in artificial soils were very similar to those of original soil, which had a more diverse microbial community, one that likely included microorganisms activated under conditions other than the anoxic conditions in the present study. This further demonstrated the high functional redundancy within the subpopulation of microorganisms that developed under the specific conditions of the present study, *i.e.*, anoxia. We were unable to evaluate the copy number of the 16S rRNA gene in artificial soils using real-time PCR because the recovery rate of DNA from soils differed, thereby disabling quantitative comparisons between the different treatments. Quantification of the microbial population that developed in artificial soils will be needed to obtain further insights into their specific activities.

The maturation period of artificial soils did not exert a significant effect on the microbial community that developed, while humic substances had a strong impact. Taken together with the results on gas production, humic substances appear to primarily shape the microbial community composition and their influence on the activity of the established community may depend on the chemical status in artificial soil, including an association with minerals. Artificial soils with (Humus 0) and without (Humus –) added humic substances produced similar levels of CO_2_ and methane (Fig. 1), while the composition and network structure of the developed microbial communities were distinct. This result implies that humic substances are one of the key factors sustaining the functional redundancy and niche specialization of the methanogenic microbial community in wetland soils (Ji *et al.*, 2018). The markedly fewer negative correlations observed between microbes in soil with humus added (Fig. 4) suggests that humus relieved the competitive relationship of microbes by providing an additional niche and nutrients.

The addition of humic substances changed the relative abundance of the different members of the *Clostridiales* group and of methanogens (Fig. 3). In the anaerobic decomposition of organic matter in wetland rice soils, clostridia participate in the hydrolysis and fermentation of organic matter and produce methanogenic substrates (H_2_/CO_2_, acetate, and formate). In their study on rice field soil in Italy, Ji *et al.* (2018) reported the existence of different methanogenic microbial network modules, in which different groups of clostridia and methanogens contribute to the process of anaerobic decomposition, including some groups whose activities are specific for the decomposition of rice straw. In the present study, the presence of humic substances affected the relative composition of *Methanobacteriaceae* and *Methanocellaceae* (Fig. 3d) and, thus, may have influenced the module(s) of hydrogenotrophic methanogenesis, which is also supported by the predicted relative abundance of hydrogenase affected by humic substances (Fig. 6a).

The reduced dominancy of *Geobacter*-related *Desulfuromonadales* sequences was another prominent effect of humic substances on the microbial community of artificial soils. *Geobacter* may compete as iron reducers with methanogens for common electron donors (*e.g.*, acetate, H_2_) (Achtnich *et al.*, 1995) or interact syntrophically with methanogens via interspecies electron transfer (Kato *et al.*, 2012), thereby playing an important role in the methanogenic decomposition of organic matter in rice field soils. Humic substances acting as an electron shuttle stimulate the growth of *Geobacter* by enhancing electron transfer between iron oxides and microbial cells (Lovley *et al.*, 1996; Zhou *et al.*, 2014). However, reduced forms of humic substances have the ability to reduce different forms of Fe(III) (Bauer and Kappler, 2009), which may limit its availability for anaerobic respiration by iron-reducing microorganisms. Fermenting bacteria were previously shown to channel electrons to ferric iron via humic acids (Straub, Benz and Schink, 2001 and references therein). Humic substances also function as electron accepters, enhancing the anaerobic decomposition of organic material in wetland soils (Keller *et al.*, 2009) and favoring microorganisms that compete with *Geobacter* for electrons because the reduction potential range of anaerobic respiration using humic substances overlaps with that of iron-based respiration (Klüpfel *et al.*, 2014). Thus, the smaller contribution of *Geobacter*-related sequences in humus-amended samples suggests that humic substances alter the mode of electron transfer in anaerobic microbial processes and that different microbial guilds adapt to the environmental changes imposed by humic substances. Recent findings support this scenario (Light *et al.*, 2018), showing that diverse species of *Firmicutes*, including *Clostridium*, which dominate humus-amended samples, possess orthologues of the genes responsible for the extracellular electron transfer driving the reduction of ferric iron.

In the present study, we successfully created artificial soils that reproduce anaerobic methane production by rice field soil, in which the maturation of matrix components was a key factor for activated microbial metabolism. The usefulness of the developed system to study the effects of soil components on the microbial community was demonstrated by examining the impact of humic substances as a case study. Further studies on the effects of each humic substance as it occurs in soil, in representative amounts (Watanabe and Kuwatsuka, 1991) and according to its chemical properties (Watanabe and Kuwatsuka, 1992; Watanabe *et al.*, 2004), are needed in order to elucidate the functions of humic substances in soil ecosystems. The approach used in the present study may also be appropriate in investigations on the roles of other soil constituents, including biological ones, such as in presence/absence and substitution experiments. Moreover, upsizing the artificial soil system may enable us to study plant–microbe interactions in soils affected by different soil biotic and abiotic factors.

## Citation

Maeda, Y., Mise, K., Iwasaki, W., Watanabe, A., Asakawa, S., Asiloglu, R., and Murase, J. (2020) Invention of Artificial Rice Field Soil: A Tool to Study the Effect of Soil Components on the Activity and Community of Microorganisms Involved in Anaerobic Organic Matter Decomposition. *Microbes Environ ***35**: ME20093.

https://doi.org/10.1264/jsme2.ME20093

## Supplementary Material

Supplementary Material
