# Supplementary material for: Invention of Artificial Rice Field Soil: A Tool to Study the Effect of Soil Components on the Activity and Community of Microorganisms Involved in Anaerobic Organic Matter Decomposition
Source: Microbes Environ. 2020 Sep 19;35(4):ME20093. doi: 10.1264/jsme2.ME20093 (PMC7734398; doi:10.1264/jsme2.ME20093)
Supplement: Supplementary file 1 — Supplementary Material [file 35_20093_s1.pdf]

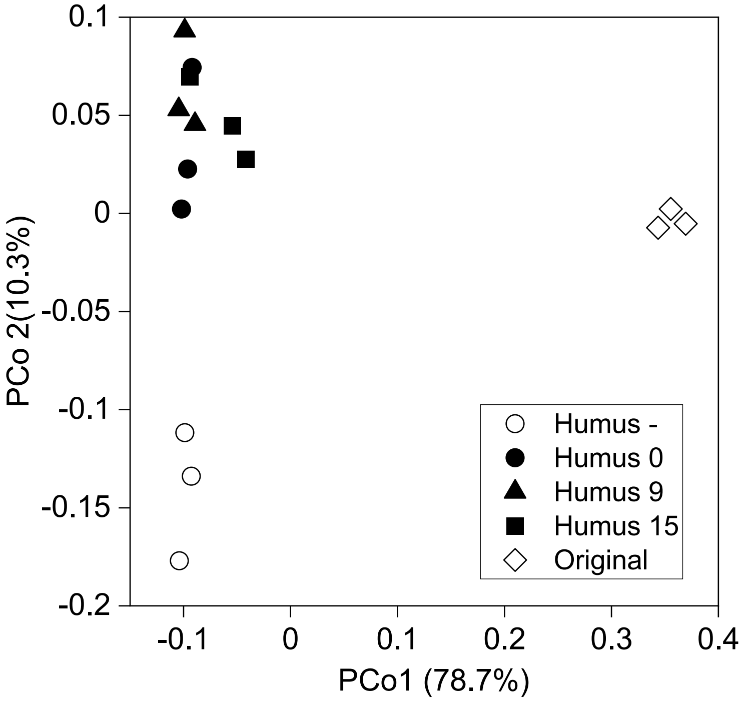

Fig.S1. Principal coordinate analysis (PCoA) based on weighted UniFrac distances of the microbial communities developed in the end of the incubation experiment.

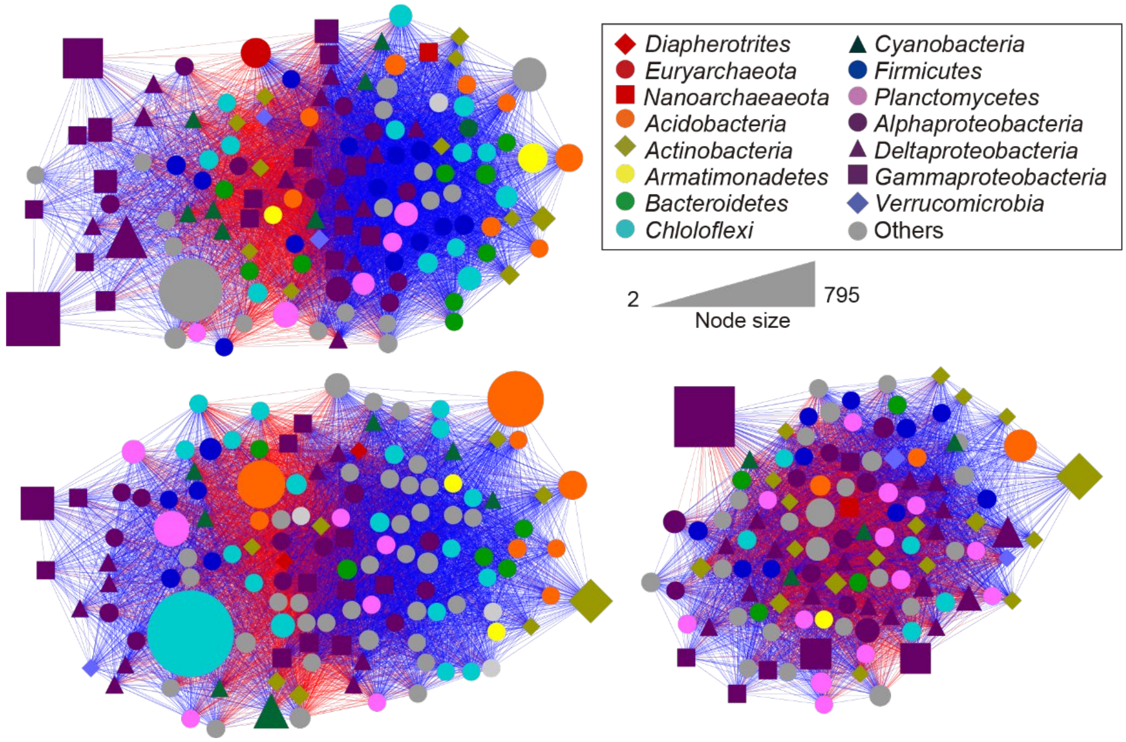

Fig. S2. Microbial co-occurrence networks based on the correlation analysis from taxonomic profiles of prokaryotic communities of the original rice soils after the incubation. The node sizes indicate the mean taxonomic abundances. Positive co-occurrence correlations ($\rho = 1$, $p < 0.05$) were indicated with blue-colored edges, while negative co-occurrence correlations ($\rho = -1$, $p < 0.05$) were indicated with red-colored edges.

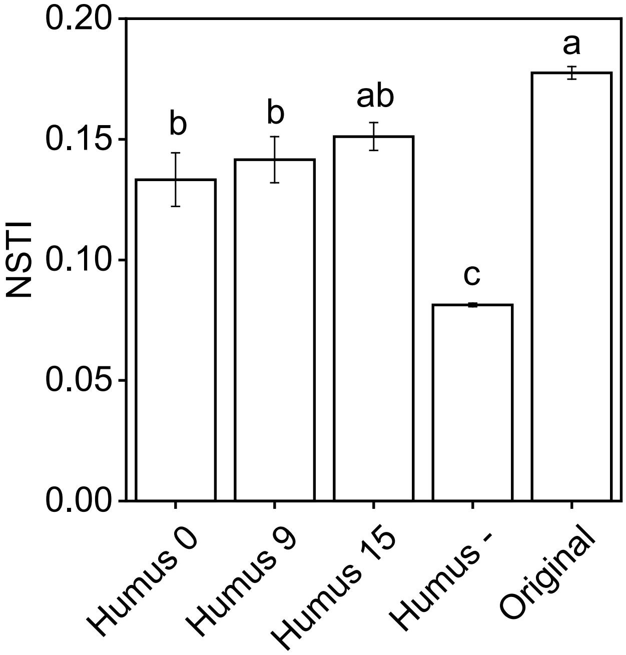

Fig.S3. Nearest sequence taxon index (NSTI) of each prokaryotic community.

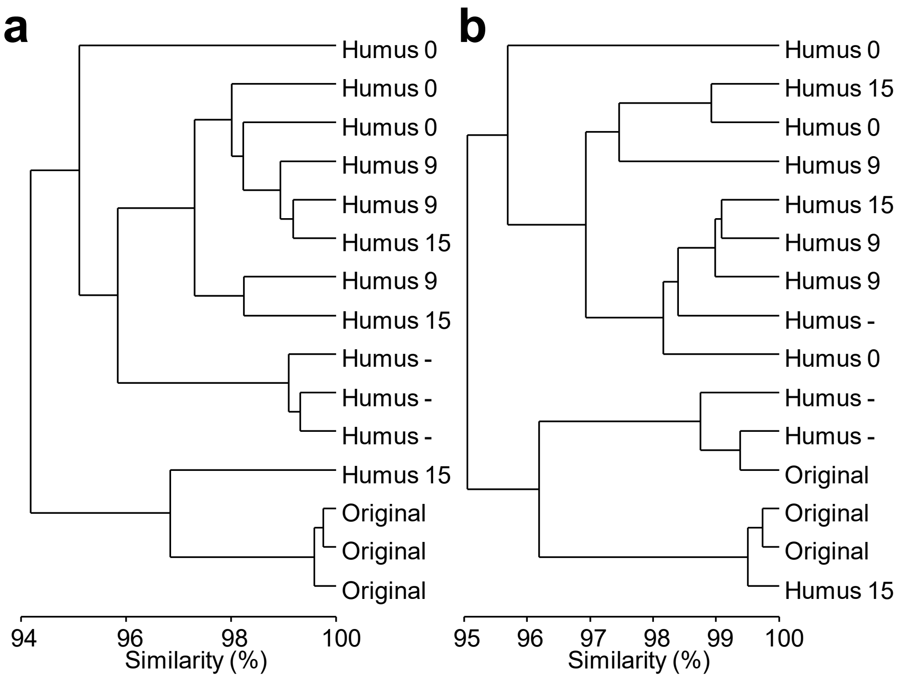

Fig.S4. Cluster analysis of the profiles of (a) the KEGG pathways and (b) COG of the artificial soils predicted from the 16S amplicon sequence data. The similarity is presented as the group average.
